# Supplementary figures and images for: Insight into the Antifungal Effects of Propolis and Carnosic Acid—Extension to the Pathogenic Yeast Candida glabrata: New Propolis Fractionation and Potential Synergistic Applications
Source: J Fungi (Basel). 2023 Apr 4;9(4):442. doi: 10.3390/jof9040442 (PMC10143237; doi:10.3390/jof9040442)

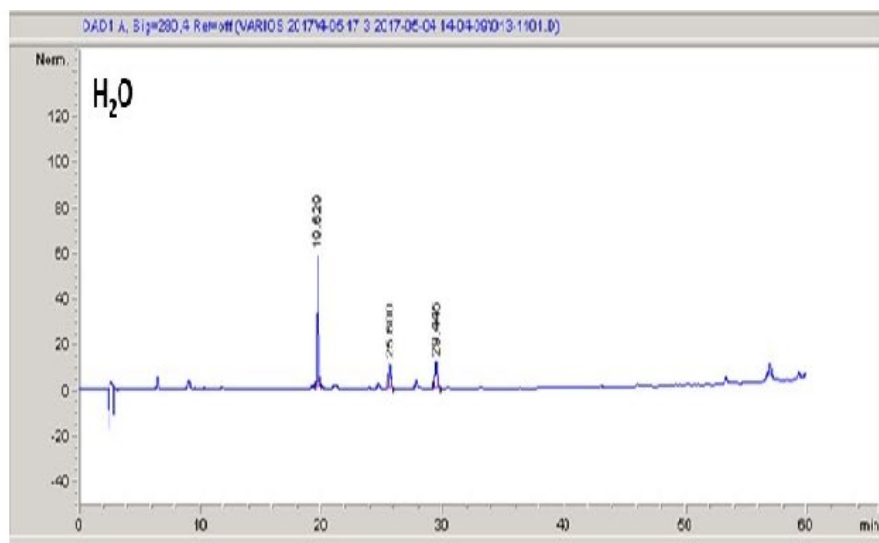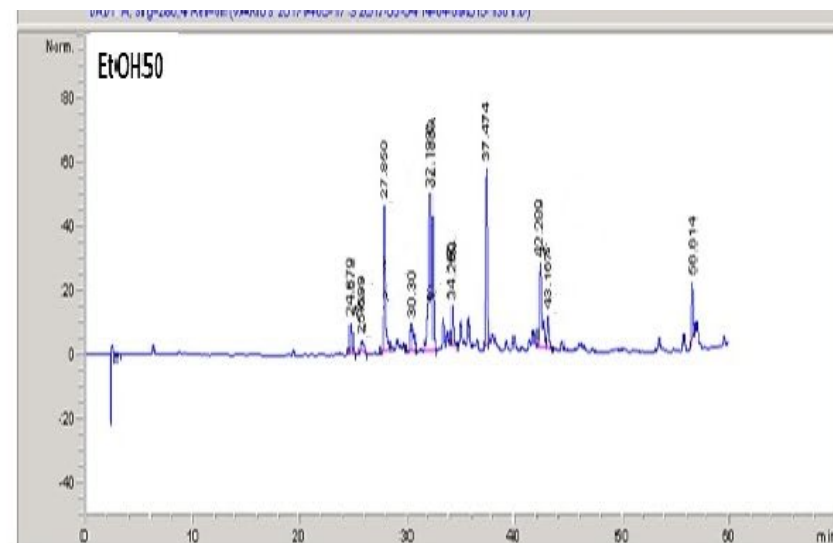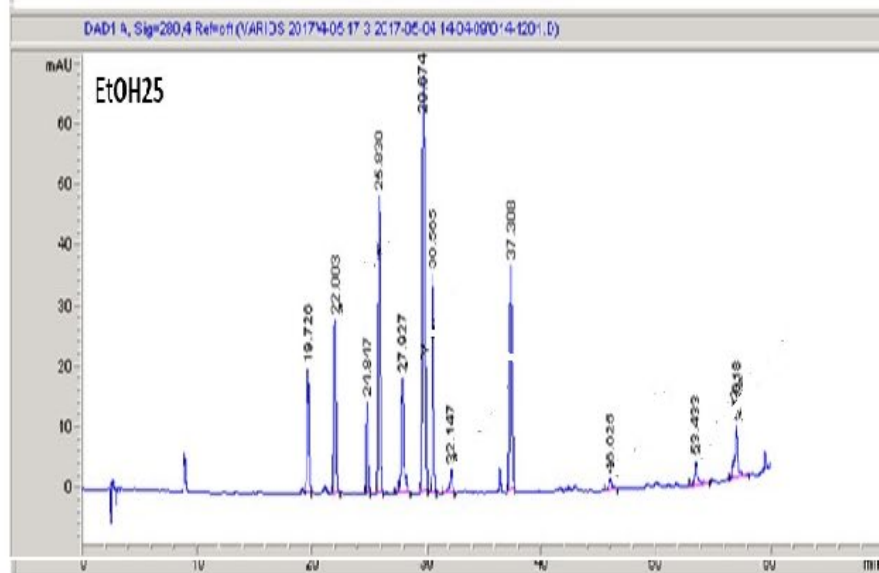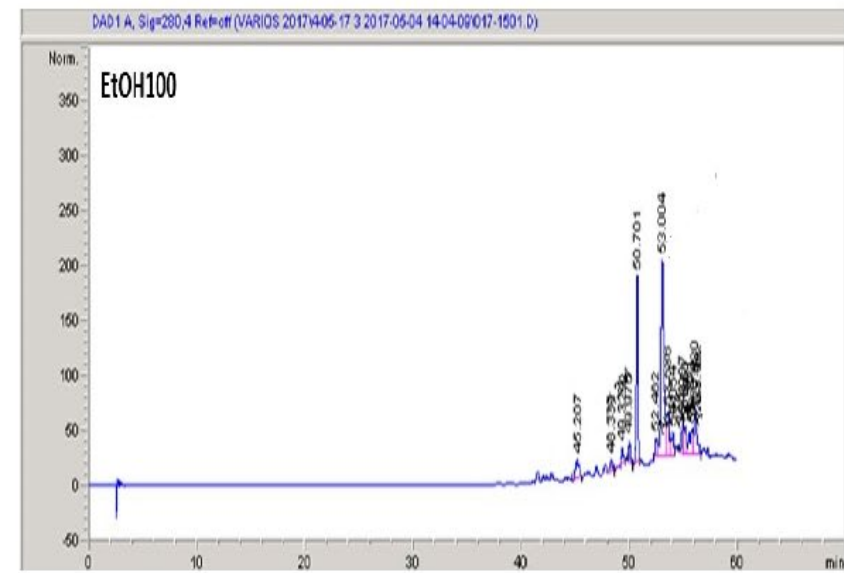

Supplement: Supplementary file 1 [file jof-09-00442-s001.zip › Figure S1.pdf]
